# Bmp7 Functions via a Polarity Mechanism to Promote Cloacal Septation

**DOI:** 10.1371/journal.pone.0029372

**Published:** 2012-01-13

**Authors:** Kun Xu, Xinyu Wu, Ellen Shapiro, Honging Huang, Lixia Zhang, Duane Hickling, Yan Deng, Peng Lee, Juan Li, Herbert Lepor, Irina Grishina

**Affiliations:** 1 Department of Urology, School of Medicine, New York University, New York, New York, United States of America; 2 Department of Toxicology, Jilin University, Changchun City, China; 3 Department of Pathology, School of Medicine, New York University, New York, New York, United States of America; 4 Department of Pathology, Memorial Sloan-Kettering Cancer Center, New York, New York, United States of America; 5 Microscopy Core, School of Medicine, New York University, New York, New York, United States of America; National Cancer Institute, United States of America

## Abstract

**Background:**

During normal development in human and other placental mammals, the embryonic cloacal cavity separates along the axial longitudinal plane to give rise to the urethral system, ventrally, and the rectum, dorsally. Defects in cloacal development are very common and present clinically as a rectourethral fistula in about 1 in 5,000 live human births. Yet, the cellular mechanisms of cloacal septation remain poorly understood.

**Methodology/Principal Findings:**

We previously detected *Bone morphogenetic protein 7 (Bmp7)* expression in the urorectal mesenchyme (URM), and have shown that loss of Bmp7 function results in the arrest of cloacal septation. Here, we present evidence that cloacal partitioning is driven by Bmp7 signaling in the cloacal endoderm. We performed TUNEL and immunofluorescent analysis on cloacal sections from *Bmp7* null and control littermate embryos. We found that loss of *Bmp7* results in a dramatic decrease in the endoderm survival and a delay in differentiation. We used immunological methods to show that Bmp7 functions by activating the c-Jun N-terminal kinase (JNK) pathway. We carried out confocal and 3D imaging analysis of mitotic chromosome bundles to show that during normal septation cells in the cloacal endoderm divide predominantly in the apical-basal direction. Loss of Bmp7/JNK signaling results in randomization of mitotic angles in the cloacal endoderm. We also conducted immunohistochemical analysis of human fetal sections to show that BMP/phospho-SMAD and JNK pathways function in the human cloacal region similar as in the mouse.

**Conclusion/Significance:**

Our results strongly indicate that Bmp7/JNK signaling regulates remodeling of the cloacal endoderm resulting in a topological separation of the urinary and digestive systems. Our study points to the importance of Bmp and JNK signaling in cloacal development and rectourethral malformations.

## Introduction

**Figure 1:**
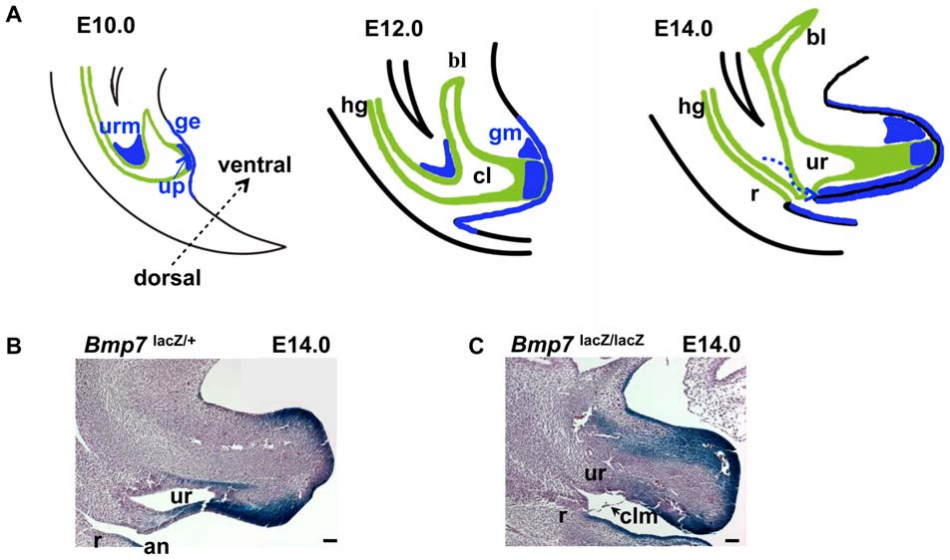
Time-line of normal cloacal partitioning in the mouse (A), and arrest in cloacal septation in *Bmp7* null embryo (C compare to B). (A) Domains of *Bmp7* expression in the urorectal mesenchyme (URM), urethral plate (up), and genital ectoderm (ge) are shown in blue. Cloacal endoderm is in green. At E10, the ventral part of the cloaca (cl) extends rostrally to give rise to the embryonic urogenital sinus and the primordium of the bladder (bl). At E12, *Bmp7* expression appears in the dorsal genital mesenchyme (gm). At E14, *Bmp7* expression in the URM shifts to the ventral portion of the genital tubercle (dashed arrow). (B, C) Histological sagittal sections of normal *Bmp7^lacZ/+^* (B) and *Bmp7^lacZ/lacZ^* null embryos (C) stained with X-gal. Heterozygous embryo (B) shows normal position of the rectum (r) and anus (an). In *Bmp7^lacZ/lacZ^* null embryo (C), a hypoplastic rectum and urethra (ur) open into a common cloacal orifice covered by the cloacal membrane (clm). Scale bars: 100 µm.

Most vertebrate phyla, including fishes, reptiles and birds, retain a common opening for the urinary tract and digestive system, called the cloaca. The cloaca is also present in the early mammalian species [1]. In contrast, marsupial and placental mammals develop separate anal and urethral openings [2], [3]. Anorectal malformations are very common in all mammals, and are part of a larger group of congenital abnormalities described as sirenomelia and caudal regression syndrome [4], [5]. In humans, mild defects in cloacal development result in a rectourethral fistula and malformed genitalia with a frequency of approximately 1∶5,000 live births. More severe defects present as a persistent cloaca or cloacal exstrophy in 1∶30,000–50,000 live births [6], [7], [8] [9], [10], [11]. The cloaca contains tissues from all embryonic cell layers: the endodermal epithelium of the hindgut (Fig. 1A, green), the cloacal membrane composed of the endoderm and ventral ectoderm, and the pericloacal mesodermal mesenchyme [12], [13], [14], [15], [16], [17]. The mesenchymal environment of the cloaca is essential for its formation and morphogenesis, and depends on sequential function of the Bone morphogenetic proteins (Bmp) and their soluble antagonist, Noggin [15], [16]. At the end of gastrulation, signaling by Bmp4 and Bmp7 expressed in the caudal part of the primitive streak promotes epithelial to mesenchymal transition to supply mesodermal cells to pericloacal mesenchyme [15], [16]. Posterior elongation of the hindgut and other caudal structures are also dependent on the most posterior homeotic genes (Hox13) Caudal-like (Cdx) transcription factors, and Wnt signaling [5], [18]. In particular, the caudal position of the cloaca and its attachment to the ectoderm depends on the function of non-canonical Wnt5a [18]. Wnt5a has also been detected in the cloacal endoderm of human embryos [19]. Histologicaly, cloacal cavity is defined at approximately 4 weeks of gestation (GA) in the human and at embryonic (E) day 9 in the mouse. At this stage, the hindgut endoderm attaches to the caudal remnants of the primitive streak to form the cloacal membrane [12], [13], [15]. During weeks 5 through 7 GA in humans, and E10 to E14 in the mouse, the cloaca is partitioned along the axial longitudinal plane into the ventral/urethral and dorsal/rectal compartments (Fig. 1A, and [8], [12], [14], [20]). Recent genetic studies by others and us indicate that cloacal septation depends on the paracrine signaling by the Sonic hedgehog (*Shh*) from the cloacal endoderm to the mesenchyme [12], [21], [22], and a mesenchymal signal by Bmp7 [20]. Defects in the *Shh* signaling pathway result in a range of cloacal abnormalities [23] and have been attributed to the impaired regulation of cell cycle rate at the caudal edge of the URM [21], [22]. In addition, function of the Six1-Eya1 transcription complex has been linked to survival and expansion of progenitor cells in the perineum [17]. We reported previously that loss of *Bmp7* results in the arrest of cloacal septation and defects in cell adhesion in the urethral endoderm [20]. Here, we show that signaling by Bmp7 activates the c-Jun N-terminal kinase (JNK) pathway, and regulates polarity of cell division and cell fate choice in the cloacal endoderm. Based on our data, we propose that Bmp7/JNK signaling regulates remodeling of the cloacal endoderm resulting in a topological separation of the urinary and digestive systems.

## Results

### Bmp7 signaling in the cloacal region is mediated by the JNK pathway

**Figure 2:**
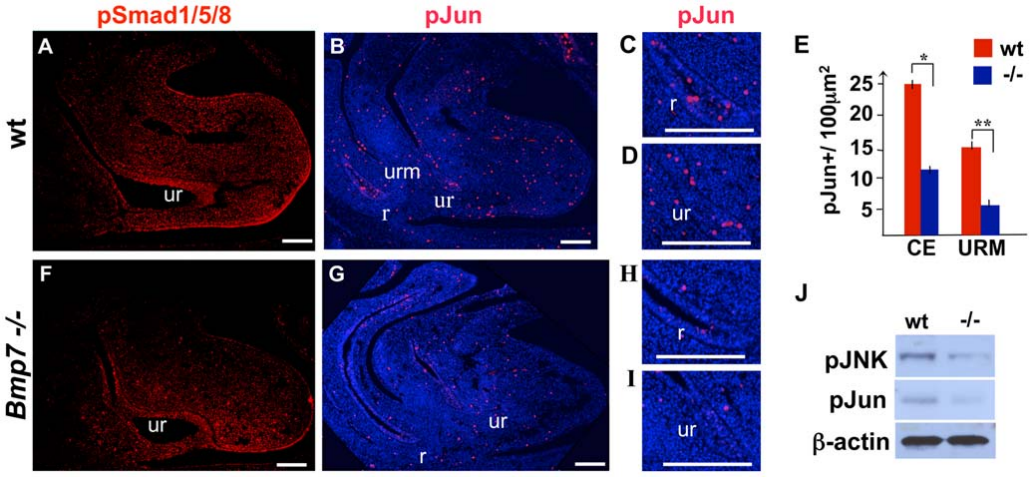
Activity of the Bmp/Smad and JNK pathways in the murine cloacal region. Wild-type and *Bmp7*-/- sagittal sections at E12.5 were immunolabeled for pSmad1/5/8 (A, F) and phospho-c-Jun (pJun) (B,G). Portions of sections labeled with pJun (B, G) are shown at high resolution: (C) wild type rectum, (D) wild type urethra, (G) *Bmp7 -/-* rectum, and (I) *Bmp7 -/-* urethra. Nuclei are labeled with DAPI. Scale bars: 100 µm. (E) Graphic presentation of the density of pJun+ cells in the cloacal endoderm (CE) and URM in wild-type (red bars) and mutant cloacas (blue bars). Student's t-test: *P<0.01; ** P<0.005. (J) Western analysis for phospho-JNK (pJNK) and pJun in control and *Bmp7* null cloacal tissues at E12.5. ß-actin was used as a loading control.

**Figure 3:**
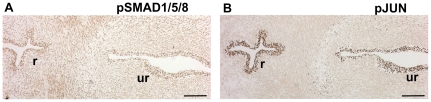
Activity of the BMP/SMAD and JNK pathways in the human fetal urethra and rectum at 7 weeks of gestation. Transverse adjacent paraffin sections are labeled for pJUN (A) and pSMAD1/5/8 (B). Scale bars: 100 µm.

We reported previously that during normal cloacal septation from E10.5 to E12.5 *Bmp7* is expressed in the URM (Fig. 1 and [20]). At cloacal stages, *Bmp7* is also expressed in the tissues of the genital appendage, including the ectoderm, urethral plate and mesenchyme (Fig. 1). Bmp signaling is commonly mediated by phosphorylation of the Smad1, 5 and 8 (Smad1/5/8) transcription factors [24], [25]. However, Bmp can also activate several types of mitogen-activated protein kinases, most notably, the JNK [26], [27]. To define the major mediators of Bmp7 signaling in the cloacal region, we first immunolabeled wild type and *Bmp7* null cloacal sections for the phosphorylated forms of Smad1/5/8 (pSmad1/5/8) (Fig. 2A, B). In E12.5 wild type cloacas, pSmad1/5/8 were detected in the ectoderm, endoderm and mesenchyme (Fig. 2A). Interestingly, *Bmp7* null cloacal endoderm, mesenchyme and ectoderm retained a significant number of pSmad1/5/8-positive cells (Fig. 2B). This remaining pSmad1/5/8 activity is likely due to the signaling by Bmp4 which is also expressed in the URM during cloacal septation [12], [28]. Next, we asked if Bmp7 signaling in the cloacal region is mediated by JNK. Immunolabeling of E12.5 wild type cloacal regions for the phosphorylated form of JNK substrate, phosho-cJun (pJun), revealed a significant number of positive cells in the endoderm of the perspective rectum (Fig. 2C), urethra (Fig. 2D) and in the URM (Fig. 2B–E). Interestingly, in *Bmp7* null embryos, pJun levels were significantly lower when compared to wild type in all cloacal compartments (Fig. 2G compare to B; H compare to C; and I compare to D). Quantitation showed that in *Bmp7* null cloacal endoderm (urethra+rectum) the number of pJun-positive (pJun+) cells was reduced by approximately 54% (P<0.01) (Fig. 2E). The number of pJun+ cells in *Bmp7* null URM was reduced by 65.0% (P<0.005). Western blot analysis confirmed significant reduction of pJun and phospho-JNK in *Bmp7* null cloacal tissues compared to wild type (Fig. 2J). These results strongly indicate that Bmp7 function in the cloacal region is mediated by the JNK pathway. In addition, we tested the levels of pSMAD1/5/8 and pJUN in the cloacal region of a human embryo just after completion of cloacal sepation at 7 weeks GA (Fig. 3). We detected pSMAD1/5/8 in the rectal and urethral endoderm and mesenchyme at low levels (Fig. 3A). In contrast, pJUN was detected at a very high levels in the rectal and urethral endoderm (Fig. 3B). These results show that both Bmp/Smad and JNK pathways function during cloacal septation in the human and mouse embryos.

### *Bmp7* is required for cell survival in the cloacal endoderm

**Figure 4:**
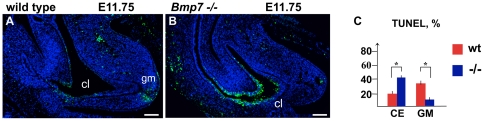
*Bmp7* functions to promote cell survival in the cloacal endoderm. (A–B) Sagittal section of E12.5 wild type (A) and *Bmp7* null (B) cloacal regions were processed for TUNEL assay, and nuclei stained with DAPI. (C) Graph presents percentage ratios of TUNEL-positive cells in the cloacal endoderm (CE) and genital mesenchyme (gm). Calculations were carried out as described in Methods. Student's t-test: *P<0.01. Scale bars: 50 µm.

During normal cloacal septation, some of the ductal and adjacent mesenchymal cells undergo programmed cell death [29]. Bmp7 is known to regulate cell survival in many organs, including the kidney and genital appendage [30], [31], [32], [33]. To determine if Bmp7 functions to regulate cell survival during cloacal septation, we carried out TUNEL analysis on wild type (Fig. 4A) and *Bmp7* null (Fig. 4B) cloacal sections at E11.75. TUNEL-positive (TUNEL+) cells were quantified (Fig. 4C) as described previously [34] (see Materials and Methods). Analysis of wild type cloacas (Fig. 4A) showed low levels of cell death in the endoderm which is consistent with a previous report [29]. We found that *Bmp7* null cloacas have a significant increase of TUNEL+ cells in the endoderm (Fig. 4B) compared to littermate controls (Fig. 4A). Quantitation uncovered that in the *Bmp7* null endoderm programmed cell death is increased by 52% (P<0.01) when compared to wild type littermates (Fig. 4C). This increase in cell death specifically affected the endoderm and not the mesenchyme (Fig. 4). Thus, cloacal endoderm is dependent on Bmp7 signal for cell survival.

### *Bmp7* promotes cell proliferation in the cloacal endoderm and mesenchyme

**Figure 5:**
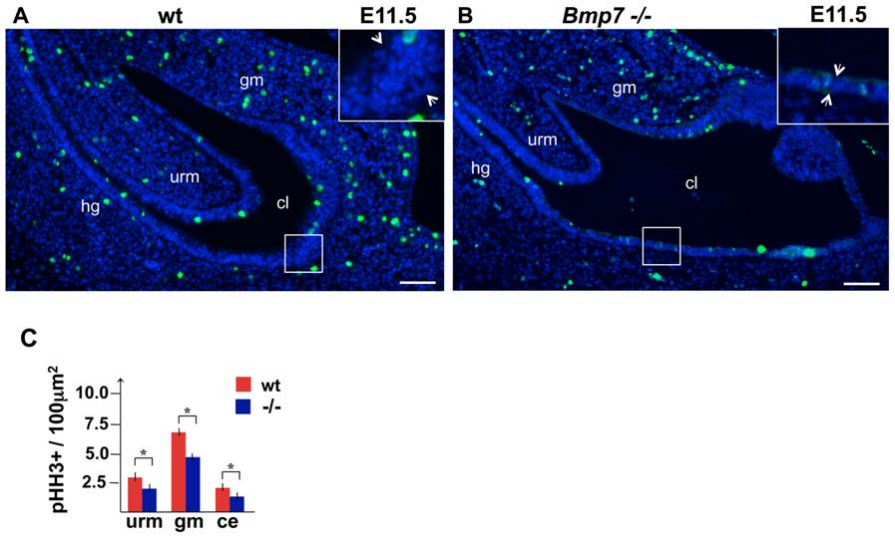
Loss of *Bmp7* results in a decrease in cell proliferation in the cloacal regions, and defects in epithelial stratification. (A, B) Sagittal sections of wild type (A) and *Bmp7* null (B) cloacal regions at E11.5 were immunolabed for pHH3, and nuclei stained with DAPI. Scale bars, 50 µm. Framed areas of the cloacal endoderm are shown at high magnification in the insets. Arrows indicate the width of the cloacal epithelium. (C) Graph presents the average number of pHH3+ cells in a 100 µm^2^ square in the cloacal endoderm (ce), URM and genital mesenchyme (gm). Student's t-test: *P<0.01.

During kidney development, Bmp7 rapidly activates the JNK pathway in the metanephric mesenchyme to promote cell proliferation [27]. To determine whether Bmp7/JNK signaling functions to promote cell proliferation in the cloacal region, we labeled wild type and *Bmp7* null cloacal sections at E11.5 for a mitotic marker, phospho-histone H3 (pHH3) (Fig. 5A, B). We compared the density of mitotic cells in wild type and mutant endoderm, URM and genital mesenchyme (Fig. 5C). In *Bmp7* null endoderm the number of mitotic cells was 34% (P<0.01) lower than in wild type endoderm. In *Bmp7* null URM and gential mesenchyme cell proliferation was 30% (P<0.01) and 31% lower (P<0.01) then in controls (Fig. 5C). These results demonstrate that Bmp7 functions to promote cell proliferation in the cloacal endoderm and mesenchyme.

### Polarity of cell divisions is disrupted in *Bmp7* null cloacal endoderm

**Figure 6:**
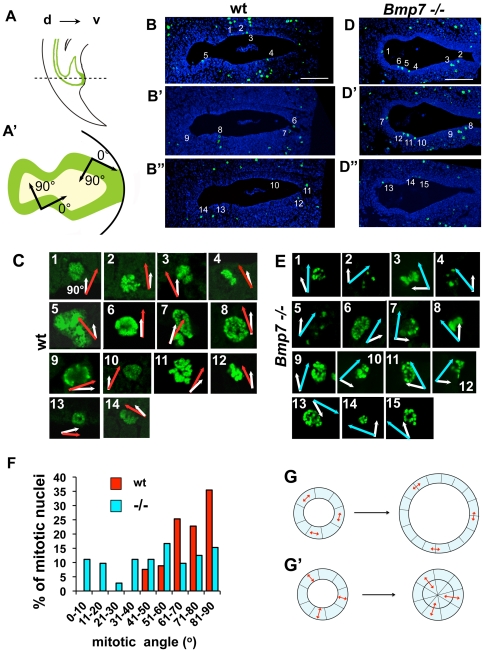
Polarity of cell divisions is disrupted in *Bmp7* null the cloacal endoderm. Sagittal (A) and transverse (A′) views of murine cloacal region at E11.5. Dashed line in (A) indicates the plane of sections in (B–B″, D–D″). Dorsal, d, and ventral, v. (A′) Radial, apical-basal, direction in the cloacal cavity is defined as 90 degrees, and tangential direction, as zero degrees. Examples of transverse cloacal sections at E11.5 of wild-type (B–B″) and *Bmp7* null (D–D″) embryos labeled for pHH3 and imaged using confocal microscopy. Scale bars, 100 µm. (D, F) Images of individual wild type (D) and *Bmp7* null (F) mitotic nuclear pairs numbered in C–C″ and E–E″ shown at 63× resolution. White vectors indicate the radial direction. Red vectors in (D) and blue vectors in (F) indicate the direction of separation of the chromosome bundles determined on confocal z-sections. (G) Graphic presentation of the distributions of mitotic angles in wild type (red bars) and *Bmp7* null (blue bars) cloacal endoderm. KS test: Nwt = 172, Nmut = 168, P<0.001. (G, G′) A simple model of the axial longitudinal section of the cloaca is depicted as a circle, one cell wide and eight cells in diameter. (G) If cell divisions are oriented tangentially (double arrows), after 8 divisions the width of the epithelium remains the same and the diameter of the lumen is increased by 1.7. (G′) If cell divisions are oriented radially, after 8 divisions, daughter cells fill the section and topologically separate dorsal and ventral lumens.

**Figure 7:**
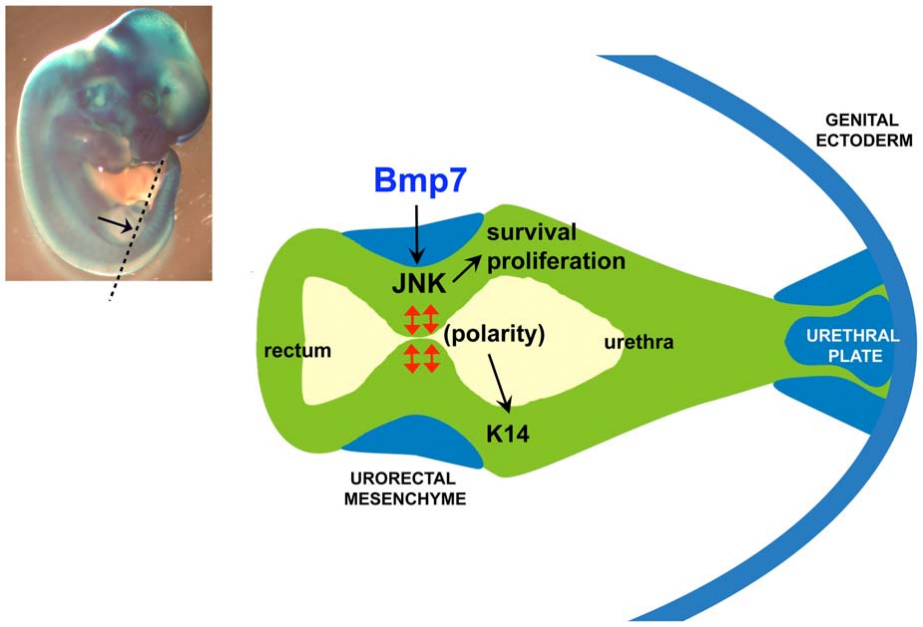
Model for cloacal septation. (A) E11.5 *Bmp7^lacZ/+^* embryo stained with X-gal. Position of the cloacal section depicted in (B) is indicated with dashed line. (B) Signaling by Bmp7 from the URM (blue) activates JNK in the cloacal endoderm. Bmp7/JNK activity promotes cell survival, proliferation and apical-basal polarity of cell divisions in the endoderm (red arrows) which facilitates topological separation of the rectum and urethra. Apical-basal polarity of cell divisions is required for epithelial differentiation and expression of K14.

In *Bmp7* null embryos, arrest in cloacal septation manifests as an enlargement of the cloacal cavity at stages E11.5 and E12.5 ([20] and Fig. 5B). We determined that the rate of cell proliferation in *Bmp7* null endoderm is decreased compared to wild type (Fig. 5C). Thus, the enlargement of *Bmp7* null cloacal lumen is not a result of increased number of cell divisions. JNKs are known to promote cell polarity and polarity of mitotic divisions by stabilizing the microtubule scaffold [35], [36]. We hypothesized that abnormal extension of the *Bmp7* null cloacal endoderm can result from the loss of JNK function leading to a disruption in the orientation of cell divisions. To test this, we examined the orientation of cell divisions in wild type and in *Bmp7* null cloacal endoderm at the mid-point of cloacal septation at E11.5 (Fig. 6A–F), and at a later septum stage at E12.5 (Fig. S1). Transverse sections of wild type (Fig. 6B–B″) and *Bmp7* null (Fig. 6D–D″) cloacas were labeled for pHH3 and imaged via confocal microscopy. Individual mitotic nuclei were identified and numbered at 10× resolution (Fig. 6B–B″ and D–D″), and mitotic angles were measured on confocal z-sections at 63× resolution (Fig. 6C and E). The apical-basal direction was defined at 90 degrees (Fig. 6A′), and is indicated with white vectors on the images of mitotic nuclei (Figs. 6C and E). Orientation of cell division was defined as the direction perpendicular to the separation of the mitotic chromosome bundles. Values of mitotic angles in wild type endoderm are indicated by red vectors (Fig. 6C). Mitotic angles in *Bmp7* null endoderm are indicated with blue vectors (Fig. 6E). Mitotic angles were calculated using Volocity 5.3.1 software (Improvision Inc., Waltham, MA). Mitotic divisions were analyzed on 20 cloacal sections, 4 each from 5 embryos of each genotype, and distributions of mitotic angles were calculated based on 175 wild type nuclei and 168 *Bmp7* null nuclei (Fig. 6F). We found that in E11.5 wild type endoderm, approximately 84% of cell divisions fell within a 60 to 90 degree range (Fig. 6F, red bars). In contrast, in *Bmp7* null endoderm orientations of cell divisions were random (Fig. 6F, blue bars). Similar results were obtained at E12.5 (Fig. S1). These results indicate that during cloacal septation Bmp7 functions to promote apical-basal orientation of cell divisions (Fig. 6F and Fig. S1A). We propose that this mechanism prompts daughter cells to extend along the length of the septal lumen leading to the topological separation of the urethra and rectum (Fig. 7 and Fig. S1A–A″). This hypothesis can be illustrated using a simple analytical model (Fig. 6G). In this model, the longitudinal section of the cloaca is depicted as a circle composed of a single cell layer of eight cells at the initial time-point (Fig. 6G). We considered two extreme conditions. One, when all cells divide laterally and their mitotic spindles are oriented tangentially to the basal membrane (Fig. 6G). Second, when all cells divide in the apical-basal orientation and their spindles are oriented radially (Fig. 6G′). After eight cell divisions, lateral divisions (Fig. 6G) produced a single cell layer duct with the diameter of the lumen increased by 1.7. In contrast, apical-basal divisions (Fig. 6G′) produced daughter cells that extended into the lumen forming a septal plate prompting a topological separation of the ventral and dorsal cloacal compartments. Consistent with this model, the epithelial lining of the extended *Bmp7* null cloacas retained sizable domains of simple cuboidal epithelium (Fig. 5B and inset, compare to 6G). In contrast, wild type cloacal walls are formed by a multilayered epithelium (Fig. 5A, and inset; [14], [26]).

### *Bmp7* null endoderm shows a defect in epithelial differentiation

**Figure 8:**
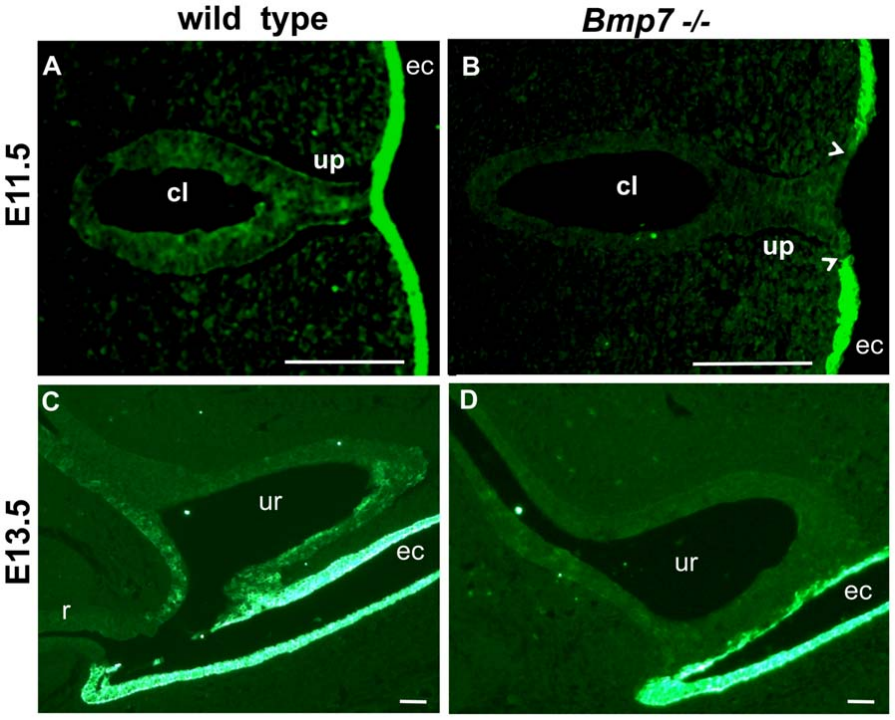
Epithelial differentiation is delayed in *Bmp7* null endoderm. Wild type and *Bmp7* null transverse cloacal sections at E11.5 (A, B) and sagittal sections at E13.5 (C, D) were labeled for Keratin 14 (K14). (A) In wild type endoderm, accumulation of K14 begins at E11.5. (B) E11.5 *Bmp7* null cloacal endoderm lacks K14. However, K14 is present in the ectoderm (ec). Arrowheads indicate the contacts between urethral plate and ectoderm. Scale bars, 100 µm.

In the ectodermal epithelium, cell fate of the progenitor cells is regulated by the polarity of cell divisions which ultimately controls stratification and terminal differentiation [37], [38], [39]. At the early stages, cloacal and urethral endoderm expresses Keratin 8 [40], [41]. Maturation of the endodermal epithelium and commitment to stratification is marked by expression and accumulation of Keratin 14 (K14) [20], [40], [41]. To determine if apical-basal polarity of cell divisions is important for differentiation of the cloacal endoderm, we examined wild type and *Bmp7* null cloacal epithelium for expression of K14 (Fig. 8). In the wild type endoderm, K14 was first detected at E11.5 (Fig. 8A). In contrast, K14 was absent from *Bmp7* null endoderm at E11.5 as well as at E13.5 (Fig. 8). Thus, similar to the ectoderm [37], apical-basal polarity of cell divisions is important for endodermal differentiation.

## Discussion

Currently, there are two sets of models describing cloacal partitioning. Models supporting morphogenetic role of the mesenchyme in cloacal septation are based on anatomical observations in human [8], histological analysis in mouse embryos [14], and genetic studies in mice deficient for the Shh pathway [21], [22]. *Shh* is expressed in the cloacal endoderm [13], and it has been suggested that Shh signals at short-range to induce faster cell divisions at the caudal edge of the URM [21], [22]. These models propose that cloacal partitioning is driven by the caudal migration [14] or proliferation of cells in the posterior portion of the URM [21], [22]. Another possibility is that Shh induces transcription of a secondary signaling molecule in the mesenchyme, such as Bmp or Wnt, which signal back to the endoderm [42], [43].

Models emphasizing morphogenetic role of the cloacal endoderm are derived from the multiple anatomical analysis of cloacal malformations in human, and in animal models, which uncovered loss of the dorsal cloacal tissue [9], [10], [11], [20], [44], [45]. In turn, our analysis provides strong evidence that cloacal partitioning is driven by reorganization of the endodermal epithelium under the influence of paracrine Bmp7 signal from the URM. During cloacal septation, *Bmp7* is expressed in the caudal URM adjacent to the axial cloacal endoderm which forms the septal plate ([20] and Fig. 7). Loss of *Bmp7* results in a signifficant increase in programmed cell death in the dorsal cloacal endoderm resulting in atrophic rectum [20]. Bmp7 requirement for survival of the hindgut endoderm is consistent with its function in other organ systems including embryonic nephrons and metanephric mesenchyme of the kidney [27], [30], [31], [46]. A large number of anatomical studies in humans [9], [10], [11] point to the loss of the dorsal cloaca as a single common feature in rectourethral malformations. Thus, further studies are essential to evaluate the role of the Bmp-regulated cell survival in cloacal development in human.

We showed that in the cloacal region Bmp7 signal is transmitted by activation of the JNK pathway. Studies in developing neurons show that the common type II component of the Bmp7 receptor complexes, Bmp receptor 2, directly interacts with JNK and phosphorylates JNK at the cellular membrane [35], [36]. Our findings, together with studies in neurons and kidneys [27], [35], [36], strongly indicate that during these organ development JNKs (encoded by *Jnk1*, *Jnk2* and *Jnk3* genes) are important mediators of Bmp7 signaling. JNK functions by regulating various aspects of the cell division cycle, including cell proliferation, programmed cell death and polarity of cell division. The later is dependent of the ability of JNK to stabilize the microtubule scaffold [35], [36]. Our analysis, consistent with a previous report [14], shows that during normal morphogenesis, the cloacal cavity forms multilayered epithelial walls (Fig. 5) and develops a progressively larger septal plate at the axial position (Fig. 6B–B″ and Fig. S1A–A″). We further show that formation of the septal plate is directed by a mechanism favoring apical-basal orientation of cell divisions in the endoderm (Fig. 6F and G). The Bmp7 signal is a strong candidate for the upstream factor in this polarizing pathway. Loss of *Bmp7* results in a randomized orientation of endodermal cell divisions (Fig. 6F) and a widening of the cloacal duct (Figs. 5B). Based on these findings, we propose a model for cloacal septation, whereby signaling from the Bmp7/JNK pathway promotes apical-basal polarity of cell divisions in the cloacal endoderm so that daughter cells may extend along the length of the septal lumen allowing topological separation of the rectum and urethra (Fig. 7).

Similar to previous reports in ectoderm [37], our studies indicate that apical-basal orientation of cell divisions is important for differentiation of the endodermal epithelium. Inactivation of *Bmp7* results in the loss of K14 and a simplification of cloacal lining which retains cuboidal epithelium. These defects in endodermal differentiation can contribute to the scrotal hypospadia observed in *Bmp7* null males [20], [41]. First, *Bmp7* null genital urethra fails to initiate timely expression of K14 which is required for the subsequent remodeling of the ventral urethral plate during sexual differentiation in the male [40], [41]. Second, abnormalities in urethral differentiation in *Bmp7* null embryos manifest already at E11.5 in a failure to maintain the contact between the endoderm and ectoderm (Fig. 8B), and at E12.5 in a loss of adhesion in the urethral plate [20].

Another interesting finding is that in addition to the endoderm, the URM itself is a target for the Bmp7 signal. Inactivation of *Bmp7* results in a significant loss of JNK activity in the URM and genital mesenchyme. This leads to a decrease in cell proliferation consistent with a similar Bmp7/JNK function in the mesenchyme of the kidney [27]. We reported previously, that *Bmp7* null mice show pronounced defects in organogenesis of stromal genital structures [20]. *Bmp7* null male genital appendage (penis) lacks corpora cavernosum and spongiousum, and shows defects in the formation of the penile bone and branching of the genital vein [20]. Thus, it is important to carry out future studies to examine the role of the Bmp and JNK pathways in regulation of cell fate in the mesenchymal cloacal lineages in regard to cell migration, condensation and differentiation of the stromal genital structures.

In summary, we present new data on the cellular mechanisms of cloacal septation, and implicate the interaction between Bmp7 and JNK pathways in regulation of cell fate choice in the cloacal endoderm. Future studies should further define the roles of the endodermal, ectodermal and mesenchymal cell lineages in cloacal development and malformations, and determine the signals and transcription factors which pattern the perspective rectum, bladder and perineum.

## Materials and Methods

### Human tissues

All human samples were obtained and used according to a protocol approved by the New York University School of Medicine's Institutional Review Board. Informed written consent was obtained by the consulting obstetrician for all specimens. Caudal tissue specimens from human fetuses at 7 to 9 weeks of gestational age (GA) were obtained following surgical abortions performed for reasons unrelated to this investigation. GA was estimated from the date of last menstrual period as well as from sonographic measurements of crown to rump and foot length. Specimens were formalin fixed, paraffin-embedded, and serially sectioned at 5 µm.

### Mouse strains

Animal handling and care was conducted in accordance with a protocol # 070802 approved by the New York University School of Medicine's (NYUSM) Institutional Animal Care and Use Committee. Embryonic day 0.5 (E0.5) was defined as 12 pm on the first day when the vaginal plug was first detected. In addition, embryos were staged by morphological appearance using the Atlas of Mouse Development [47]. *Bmp7^lacZ^* strain and genotyping procedures have been described previously [20], [30].

### Immunolabeling

For immunostaining, human fetal tissue sections were routinely deparaffinized and hydrated, and endogenous peroxidase activity was blocked with 3% hydrogen peroxide in methanol for 15 minutes. Antigen retrieval was carried out by microwaving tissue sections at full power for 30 minutes in Antigen Unmasking Solution (catalogue number H-3300, Vector Laboratories, Inc., Burlingame, CA). Tissue sections were incubated for 1 hour at room temperature with primary antibodies to pSmad1/5/8 (catalogue number 9511, Cell Signaling, Danvers, MA) or pJun (catalogue number 9164, Cell Signaling Technology, Danvers, MA) diluted at 1∶100 in 1% normal goat serum (Gibco), then rinsed in Phosphate Buffer Saline, and incubated for 1 hour at room temperature with biotinylated goat anti-rabbit secondary antibodies (Vector Laboratories, Inc., Burlingame, CA). Sections were then rinsed, incubated with streptavidin-conjugated horseradish peroxidase (Invitrogen, Carlsbad, CA), and developed in a solution containing 3,3′-diaminobenzidine and H_2_O_2_ using the Peroxidase Substrate Kit (DAB, catalogue number SK-4100, Vector Laboratories, Inc., Burlingame, CA). Sections were then mounted, and imaged under bright light using Axiovision 2 scope equipped with a digital camera (Carl Zeiss MicroImaging, Thornwood, NY).

Immunofluorescent analysis on murine paraffin-embeded tissue sections was performed as described previously [34]. Primary antibodies for pHH3 (catalogue number 6570, Upstate Biotechnology, Billerica, MA) were used at 1∶100 dilutions, followed by Alexa Fluor secondary antibodies. Primary antibodies for K14 (catalogue number PRB-155P, Covance, Princeton, NJ), pSmad1/5/8 (catalogue number 9511, Cell Signaling, Danvers, MA) and pJun (catalogue number 9164, Cell Signaling Technology, Danvers, MA) were used at 1∶100 dilution with Tyromide Signal Amplification (TSA) Kit # 23 (Invitrogen, Carlsbad, CA) as described previously [48]. Fluorescent imaging was performed using Zeiss Axioplan3.1 fluorescent microscope at the Urology Research Laboratories, and the LSW 510 and LSW 710 confocal scopes at the New York University School of Medicine Microscopy Core.

### Western Blot analysis

Cloacal/genital regions were dissected from E12.5 embryos, and Western Blot analysis was performed by standard protocols as described previously [49]. Antibodies specific for pJun (Cell Signaling Technology) and pJNK (catalogue number 4668, Cell Signaling Technology, Danvers, MA) were used at 1∶300 dilution, and _ß_-actin (catalogue number A2228, Sigma, St. Louis, MO) at 1∶2500 dilution. Experiments were repeated three times.

### Programmed cell death

Programmed cell death was analyzed on paraffin tissue sections by terminal deoxynucleotidyl transferase dUTP nick end labeling reaction (TUNEL) assay using the Programmed Cell Death Kit and following the manufacturer's instructions (Roche Applied Science, Indianapolis, IN) as we described previously [34].

### Quantitation and statistics

For each experiment, five mutant and control embryos were analyzed for each embryonic stage. To achieve statistical significance of the collected data, counting of cells positive for particular signal was carried out on 20 sections, 4 each from 5 embryos of each genotype. Imaging was performed using Axiovision 2 fluorescent scope and software (Carl Zeiss MicroImaging, Thornwood, NY), or LSM 510 and LSM 710 confocal scopes and Volocity 5.3.1 Software (Perkin Elmer, Waltham, MA). Calculations of the percentage ratios were carried out in comparison to the total cell number in the counted compartments identified by 4′,6-diamidino-2-phenylindole (DAPI) staining of the nuclei. Calculations of the average cell densities positive for particular signal was carried out in five 10^6^ µm^2^ quadrants on 20 sections, 4 each from 5 animals of each genotype as we described [33], [23]. Statistical significance of the differences in each set of experiments was determined by Student's t-test. P<0.05 was considered significant.

### Analysis of the distribution of mitotic angles

To determine the distribution of mitotic angles in wild type and *Bmp7* null cloacal endoderm, we immunolabeled for pHH3 and imaged using the LSW 710 confocal microscope at the NYUSM Microscopy Core. Mitotic divisions were analyzed on 20 cloacal sections, 4 each from 5 embryos of each genotype, and distributions of mitotic angles were calculated for 175 wild type nuclei and 168 *Bmp7* null nuclei. Mitotic angles were defined as the direction perpendicular to the separation of the mitotic chromosome bundles. Apical-basal direction was defined at 90 degree angle. Mitotic angles were measured on confocal z-sections at 63× resolution using Volocity 5.3.1 3D Imaging software (Improvision Inc., Waltham, MA). Statistical significance of the differences between the angles distribution in wild type and mutant tissues were determined by two-sample Kolmogorov-Smirnov (KS) test [50], [51].

## Supporting Information

Figure S1 **Formation of cloacal septum. Polarity of cell divisions in E12.5 wild type and** ***Bmp7*** **null the cloacal endoderm**. Confocal imaging, angle measurements and analysis are done as in Fig. 6 and described in Materials and Methods. (A) Distributions of mitotic angles in wild type (red bars) and *Bmp7* null (blue bars) cloacal endoderm. At E12.5 73+/−2% of the wild type cell divisions fell in the 60 to 90 degree range (red bars). In *Bmp7* null cloacal endoderm, orientation of cell divisions are randomized (blue bars). KS test: n_wt_ = 170, n_mut_ = 159, P<0.0018. (B–B″ and C–C″) Transverse cloacal sections of wild-type (B–B″) and *Bmp7* null (C–C″) embryos labeled for pHH3. Scale bars, 100 µm. (C, D) Images of individual wild type (D) and *Bmp7* null (E) mitotic nuclear pairs. Nuclei are numbered from upper left corner and clockwise in B–C″. White vectors indicate radial direction. Red vectors in (D) and blue vectors in (E) indicate the value of mitotic angle.
(TIF)
