# Supplementary figures and images for: Bmp7 Functions via a Polarity Mechanism to Promote Cloacal Septation
Source: PLoS One. 2012 Jan 13;7(1):e29372. doi: 10.1371/journal.pone.0029372 (PMC3258230; doi:10.1371/journal.pone.0029372)

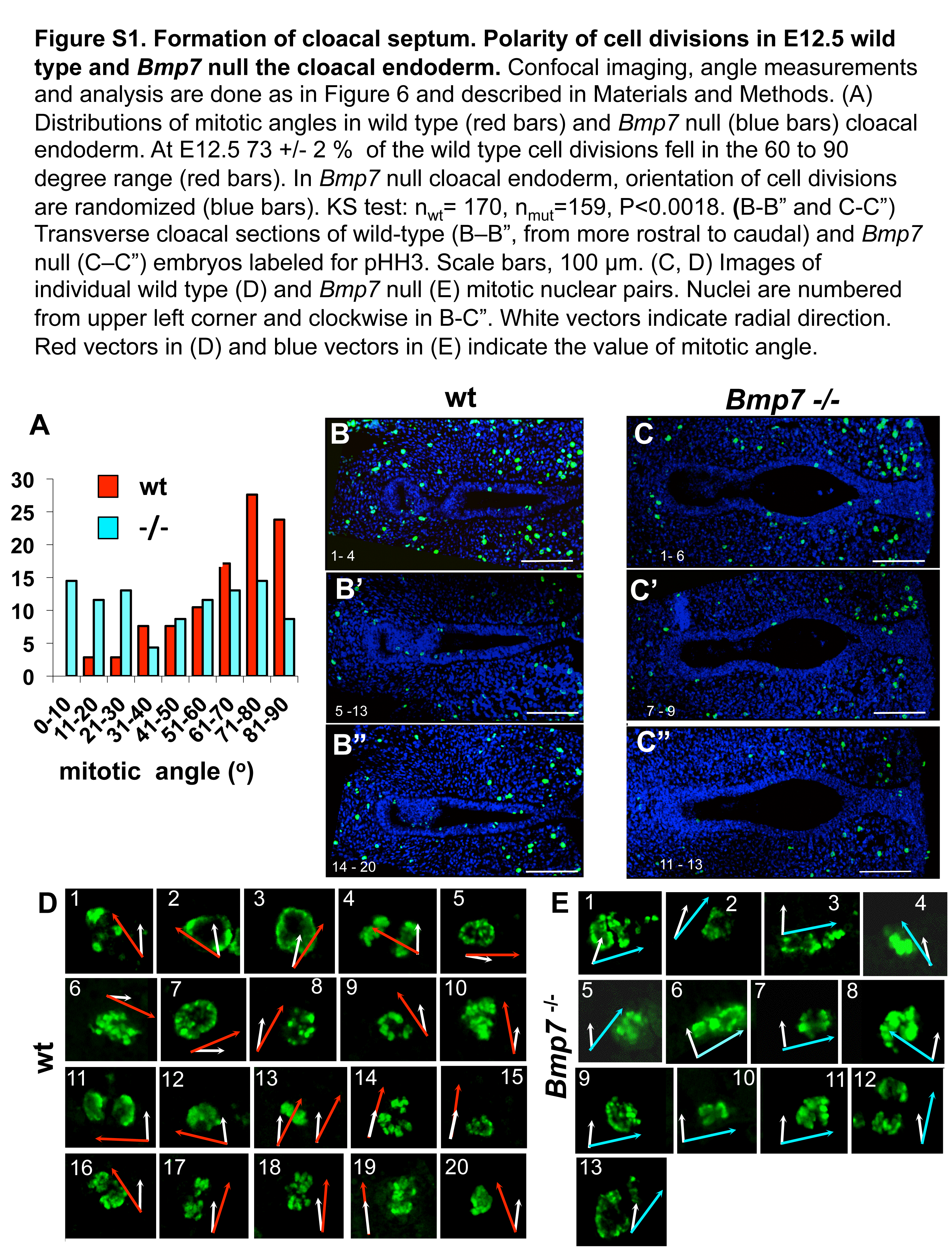

Supplement: Figure S1 — Formation of cloacal septum. Polarity of cell divisions in E12.5 wild type and Bmp7 null the cloacal endoderm. Confocal imaging, angle measurements and analysis are done as in Fig. 6 and described in Materials and Methods. (A) Distributions of mitotic angles in wild type (red bars) and Bmp7 null (blue bars) cloacal endoderm. At E12.5 73+/−2% of the wild type cell divisions fell in the 60 to 90 degree range (red bars). In Bmp7 null cloacal endoderm, orientation of cell divisions are randomized (blue bars). KS test: nwt = 170, nmut = 159, P<0.0018. (B–B″ and C–C″) Transverse cloacal sections of wild-type (B–B″) and Bmp7 null (C–C″) embryos labeled for pHH3. Scale bars, 100 µm. (C, D) Images of individual wild type (D) and Bmp7 null (E) mitotic nuclear pairs. Nuclei are numbered from upper left corner and clockwise in B–C″. White vectors indicate radial direction. Red vectors in (D) and blue vectors in (E) indicate the value of mitotic angle. (TIF) [file pone.0029372.s001.tif]
